# Cytotoxic effect of disulfiram/copper on human cervical cancer cell lines and LGR5-positive cancer stem-like cells

**DOI:** 10.1186/s12885-022-09574-5

**Published:** 2022-05-09

**Authors:** Hao-Zhe Cao, Wen-Ting Yang, Peng-Sheng Zheng

**Affiliations:** 1 Department of Reproductive Medicine, the First Affiliated Hospital of the Medical College, Xi’an Jiaotong University Medical School, 76 West Yanta Road, Xi’an, 710061 China; 2 Division of Cancer Stem Cell Research, Key Laboratory of Environment and Genes Related to Diseases, Ministry of Education, Xi’an Jiaotong University Medical School, Xi’an, 710061 China

**Keywords:** LGR5, Disulfiram, Copper, Cervical cancer, Cancer stem cell

## Abstract

**Background:**

Tumor resistance is a global challenge for tumor treatment. Cancer stem cells (CSCs) are the main population of tumor cells for drug resistance. We have reported that high aldehyde dehydrogenase (ALDH) activity represents a functional marker for cervical CSCs. Here, we aimed at disulfiram (DSF), an ALDH inhibitor, that has the potential to be used for cervical cancer treatment.

**Methods:**

MTT assay, western blot, vector construction and transfection, cell sorting and in vivo anti-tumor assays were performed using cervical cancer cell lines SiHa and HeLa. Cell cycle distribution and cell apoptosis were carried out by flow cytometry. The cytotoxicity of DSF was detected by MTT assay and cervical cancer xenograft models.

**Results:**

DSF was cytotoxic to cervical cancer cell lines in a copper (Cu)-dependent manner. Disulfiram/copper (DSF/Cu) complex induced deregulation of S-phase and inhibited the expression of stemness markers in cervical cancer cells. Furthermore, DSF/Cu could also reduce the cancer stem cell-like LGR5^+^ cells which lead to cisplatin resistance in cervical cancer cells. DSF/Cu complex had the greater antitumor efficacy on cervical cancer than cisplatin in vitro and in vivo.

**Conclusion:**

Our findings indicate that the cytotoxicity of DSF/Cu complex may be superior to cisplatin because of targeting LGR5-positive cervical cancer stem-like cells in cervical cancer. Thus, the DSF/Cu complex may represent a potential therapeutic strategy for cervical cancer patients.

**Supplementary Information:**

The online version contains supplementary material available at 10.1186/s12885-022-09574-5.

## Background

Cervical cancer is the fourth most common form of cancer among women worldwide, with the disease claiming the lives of more than 342,000 women in 2020 [1, 2]. Although HPV testing and Papanicolaou smear screening have brought great benefits to the prevention and early treatment of cervical cancer, the patients with metastasis are still facing great difficulties in the treatment. The five-year survival rate of cervical cancer patients without metastasis was over 80%, but the survival rate of metastatic patients was unfavorable and under 42% without dramatic improvement for several decades [3]. It is critical to develop a novel treatment in order to increase the overall survival rate of cervical cancer patients.

Disulfiram (DSF) has been used as a first-line anti-alcoholism drug in the world for over 60 years [4, 5]. DSF is a small molecule with a molecular weight of 296.54. Recently, accumulating evidence demonstrates that DSF is involved in inhibiting prostate cancer, lung cancer, breast cancer, liver cancer, ovarian cancer and oesophageal carcinoma cell proliferation [6–13]. DSF also potentiates the cytotoxicity of several classical anticancer drugs as well as radiation [14–18]. DSF can be rapidly reduced to form two molecules of diethyldithiocarbamate in serum, which is a strong chelator of transition divalent metal ions [19]. The anticancer effect of DSF has been shown to be copper (Cu) dependent [20–22]. DSF improves the transport of copper into cancer cells [23]. Copper plays an important role in redox reactions and triggers generation of reactive oxygen species (ROS) in the cells [24]. In addition, previous studies have reported that the DSF/Cu complex is highly cytotoxic to the cancer cells but not the normal cells [25, 26]. It is noteworthy that the repurposing of DSF to cancer treatment has received recent interest.

It has been proposed that disulfiram-loaded immediate and extended release vaginal tablets have the potential to be used for the localized treatment of cervical cancer [27, 28]. However, these studies showed that the inhibition of cervical cancer cell viability required a high dose of disulfiram and the studies did not involve the addition of copper. We have found that high aldehyde dehydrogenase (ALDH) activity represents both a functional marker for cervical cancer stem cells (CCSCs) and a target for cervical cancer therapies [29]. In addition, our previous studies demonstrated that LGR5 promotes cancer stem cell traits and chemoresistance in cervical cancer [30]. These findings led us to investigate the effect of disulfiram and copper in cervical cancer and CCSCs.

In this study, we evaluated the effects of DSF/Cu complex in cervical cancer cells by using in vitro and in vivo models. Our data suggested that the DSF/Cu complex may be a potential therapeutic drug for human cervical cancer.

## Methods

### Cell culture

The cervical cancer cell lines SiHa and HeLa were cultured in DMEM medium with L-glutamine (Invitrogen, Heidelberg, Germany) supplemented with 10% fetal bovine serum and 1% penicillin/streptomycin (both from Millipore, Darmstadt, Germany) in a humidified incubator at 37 °C and 5% CO_2_.

### MTT assay

SiHa and HeLa cells were seeded in 96-well plates at a density of 10^4^ cells/well and allowed to recover overnight before initiating the drug treatments. The cells were exposed to different doses of disulfiram (Sigma), CuCl_2_ (Sigma) and (or) 10 μmol/L cisplatin (DDP, Teva Parenteral, CA) for different times in different experiments as indicated. Cell viability was assessed using the 3-(4,5-Dimethyl-1, 3-thiazol-2-yl)- 2,5-diphenyl-2H-tetrazol-3-ium bromide (MTT; Sigma-Aldrich) assay. Following the manufacturer’s instructions, 20 μl of MTT solution was added into 200 μl of the culture media. The plates were then incubated for 4 h at 37 °C, and the optical density was measured at 490 nm.

### Assessment of apoptosis

Apoptotic status was determined by PE Annexin-V/7-AAD Apoptosis Detection Kit I (559,763, BD) using flow cytometry following the manufacturer’s instructions. Briefly, 5 × 10^5^ cells were seeded in T25 flasks for 24 h and treated with drugs for a further 48 h. The cells were rinsed with calcium-containing HBSS and detached using EDTA-free trypsin. The detached cells were washed twice with cold PBS and then resuspended in 100 μl Binding Buffer at a concentration of 1 × 10^6^ cells/ml. All cells were incubated with PE Annexin V (5 μl) and 7-AAD (5 μl) at RT for 15 min in the dark. The cells were diluted in 400 ml of Binding Buffer and then 20,000 events were acquired and analyzed by a FACSCalibur Flow Cytometry (Becton Dickinson, USA). Apoptosis and necrosis were evaluated using FL2 (Annexin V) vs FL3 (7-AAD) plots. The cells stained with Annexin V only were classified as early apoptosis and the Annexin V and 7-AAD double-stained cells were classified as late apoptosis or necrosis.

### Western blot

Cells were lysed in a lysis buffer (50 mM Tris-HCl, pH 7.4; 150 mM NaCl; 2 mM EDTA; 1% NP-40; and 0.1% sodium dodecyl sulfate) that contained a protease inhibitor cocktail (Complete Mini; Roche Diagnostics, Branchburg, NJ). The blots were cutted according to molecular size markings prior to hybridisation with antibodies. The membranes were incubated with antibodies against human Bcl2 (1:200 dilution, sc-7382, Santa Cruz Biotechnology), ABCG2 (1:50 dilution, sc-58,222, Santa Cruz Biotechnology), P53 (1:500 dilution, sc-263, Santa Cruz Biotechnology), P27^Kip1^ (1:500 dilution, sc-528, Santa Cruz Biotechnology), LGR5 (1:200 dilution, Abnova, Taipei, Taiwan) and β-actin (1:1000 dilution, sc-47,778, Santa Cruz Biotechnology) at 4 °C overnight followed by incubation with horseradish peroxidase-conjugated secondary immunoglobulin G (IgG; Thermo Fisher Scientific, New York, NY). The membranes were briefly incubated with an enhanced chemiluminescence reagent (Millipore, Billerica, Mass) and then visualized on X-ray films [31].

### Cell cycle analysis

Analysis of cell cycle progression was performed using DNA staining. Briefly, cervical cancer cells were plated into T-75 flasks at an initial seeding density of 10^6^ cells/flask. After 48 h treatment, trypsinized cells were washed twice with cold PBS, followed by fixation with ice-cold 70% ethanol overnight at 4 °C. After washing twice with PBS, the cells were incubated with 50 μg/ml propidium iodide (Sigma-Aldrich, St. Louis, MO, USA) and 10 μg/ml RNaseA (Sigma) for 30 min at room temperature. The cells were then analyzed using a FACSCalibur (Becton Dickinson, USA) with the FlowJo software (Tree Star Inc., Ashland, USA).

### Immunocytochemistry

For immunocytochemistry, cells were plated on cover slips, fixed with 4% paraformaldehyde for 30 min at room temperature, and permeabilized with 0.2% Triton X-100 for 20 min at room temperature. The slides were incubated with a rat monoclonal antibody raised against human LGR5 (1:50, Abnova, Taipei, Taiwan) overnight at 4 °C and then with secondary antibodies for 30 min at room temperature followed by diaminobenzidine development. All slides were examined under an Olympus-CX31 microscope and the images were acquired by DP Controller (Olympus, Tokyo, Japan) [30].

### Flow cytometry analysis and FACS isolation of cells

The ALDH enzymatic activity of the cells was measured using the ALDEFLUOR kit (Stem Cell Technologies, Vancouver, BC, Canada), according to the manufacturer’s instructions. The brightly fluorescent ALDH-expressing cells were detected using a FACSCalibur flow cytometer (BD Biosciences). As a negative control, cells were stained under identical conditions after treatment with the specific ALDH inhibitor diethylaminobenzaldehyde (DEAB) [29]. The expression of LGR5 and CD49f in cervical cancer cells was measured using the Alexa Fluor® 647 Rat anti-Human LGR5 (N-Terminal) antibody (562,903, BD) and PE Rat anti-Human CD49f antibody (555,736, BD) according to the manufacturer’s instructions. The data were analyzed using the CellQuest program (BD Biosciences).

### Vector construction and transfection

Human full-length LGR5 cDNA was amplified by reverse transcription polymerase chain reaction using mRNA extracted from SW620 cells. The primer sequences were designed as follows:

F5′-CTTCTCGAGCTACTTCGGGCACCATGG AC-3′; and.

R5′-GCGGGTACCTTAGAGACATGGGACAAA TG-3′.

The LGR5 DNA fragment was subsequently cloned into the XhoI and SmaI sites of the pCAG-AcGFP vector (Clontech, Mountain View, CA, USA) to generate the pCAG-AcGFP-LGR5 recombinant plasmid. The LGR5-overexpression vectors were transfected into SiHa and HeLa cells using the Lipofectamine 2000 reagent (Invitrogen, Carlsbad, CA, USA) according to the manufacturer’s protocol. The transfected cells were treated with G418 (Calbiochem, La Jolla, CA, USA) for 3 weeks, and drug-resistant colonies were collected, expanded, and identified [30].

### In vivo anti-tumor assays

The cervical cancer bearing BALB/c nude mice model was used to investigate the in vivo antitumor efficacy of disulfiram/copper complex. Female BALB/c-nude mice (6–7 weeks old) were obtained from Shanghai SLAC Laboratory Animal Co.,Ltd. (Shanghai, China) and housed in a SPF room that was maintained at a constant temperature (22 °C–25 °C) and humidity (40–50%). The sorted tumor cells (1 × 10^6^) were resuspended in 200 μl of 1:1 PBS/Matrigel (BD Biosciences) solution and injected into the subcutis of the two flanks on the dorsum of each mouse (eight mice in each group). Drug treatment (DSF 30 mg/kg, CuCl_2_ 1.5 mg/kg, DDP 5 mg/kg, 0.9% saline) was initiated after 3 days of cell injection and administered via intraperitoneally injection twice weekly. DSF (6 mg/ml stock) was prepared as follows: 120 mg DSF was dissolved in 1 ml DMSO, made up to 10 ml with PEG300 (Selleck), then added to 10 ml H_2_O, vortexed and filter sterilized. Animals had access to food and water ad libitum. The tumor size was measured using a vernier caliper every week, and the volume was calculated with the following formula: V = (length×width^2^) /2.

### Statistical analysis

Statistical analyses were performed using the GraphPad Prism 8.00 software (La Jolla, CA, USA). In the comparisons of 2 groups, Student’s t-test was used to determine statistical significance. To examine differences among > 2 groups, non-parametrical Kruskal–Wallis test or one-way analysis of variance (ANOVA) was used to determine statistical significance. Kaplan-Meier survival analysis was performed, and survival curve comparisons were performed using the log-rank (Mantel-Cox) test. *P* values of ≤0.05 were regarded as statistically significant.

## Results

### DSF and copper produce synergistic cytotoxicity in cervical cancer cell lines

**Fig. 1:**
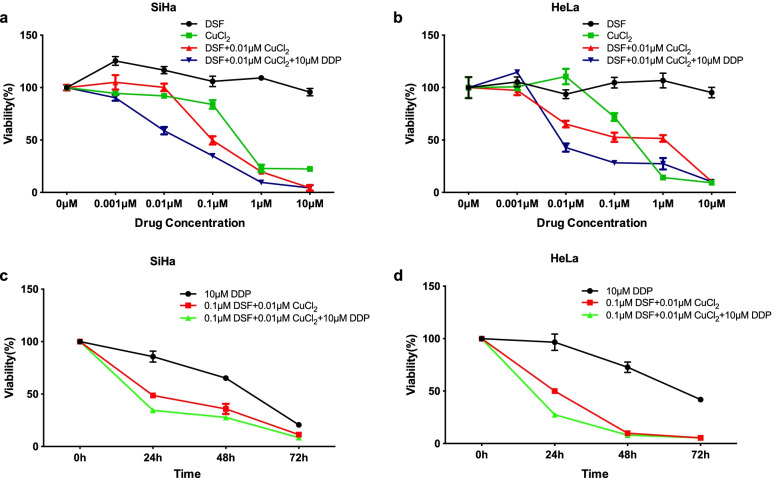
Inhibition of cervical cancer cell proliferation by disulfiram/copper complex. **a**-**b** SiHa and HeLa cells were treated with different concentrations of disulfiram and CuCl_2_ alone or in combination with or without 10 μM DDP for 24 h, after which their cell viabilities were determined by the MTT assay. **c**-**d** SiHa and HeLa cells were treated at different time points (0, 24, 48, and 72 h) with a constant low-dose of disulfiram/copper complex compared with DDP alone or in combination, after which their cell viabilities were determined by the MTT assay. Data represent mean ± S.D. of triplicate experiments

Initially, cell viability assays were conducted to investigate the sensitivity of a panel of cervical cancer cell lines to disulfiram. SiHa and HeLa cell lines were treated with different concentrations of DSF or copper (CuCl_2_, Cu) alone or in combination followed by MTT assay. No cytotoxicity was observed in two cervical cancer cell lines until the cells were treated with 10 μM disulfiram or 0.01 μM CuCl_2_ alone (*P* > 0.05). However, in the presence of 0.01 μM CuCl_2_, as low as 0.1 μM disulfiram showed strong cytotoxicity in SiHa cells and 0.01 μM disulfiram in HeLa cells (both at *P* < 0.001). The addition of 10 μM cisplatin significantly improved the cytotoxicity of DSF/Cu complex at concentrations ranged from 0.001 μM to 0.1 μM DSF in SiHa cells and 0.01 μM to 1 μM DSF in HeLa cells (*P* < 0.05, Fig. 1a-b).

To further investigate the synergistic cytotoxicity of DSF/Cu complex, the viability of SiHa and HeLa cells was measured after treatment with a constant low-dose of DSF/Cu compared with 10 μM DDP alone or in combination for different incubation times. The cytotoxicity of DSF/Cu was significantly stronger than DDP alone at all different time points in both cell lines (24 h, 48 h, 72 h; all at *P* < 0.01). DSF/Cu complex combined with DDP showed significantly greater cytotoxicity than DSF/Cu only at the time point of 24 h in two cancer cell lines (*P* < 0.01, Fig. 1c-d). These data showed that DSF/Cu complex alone or combinated with DDP can produce appreciable cytotoxicity in cervical cancer cells.

### DSF/cu complex induces apoptosis in cervical cancer cell lines

**Fig. 2:**
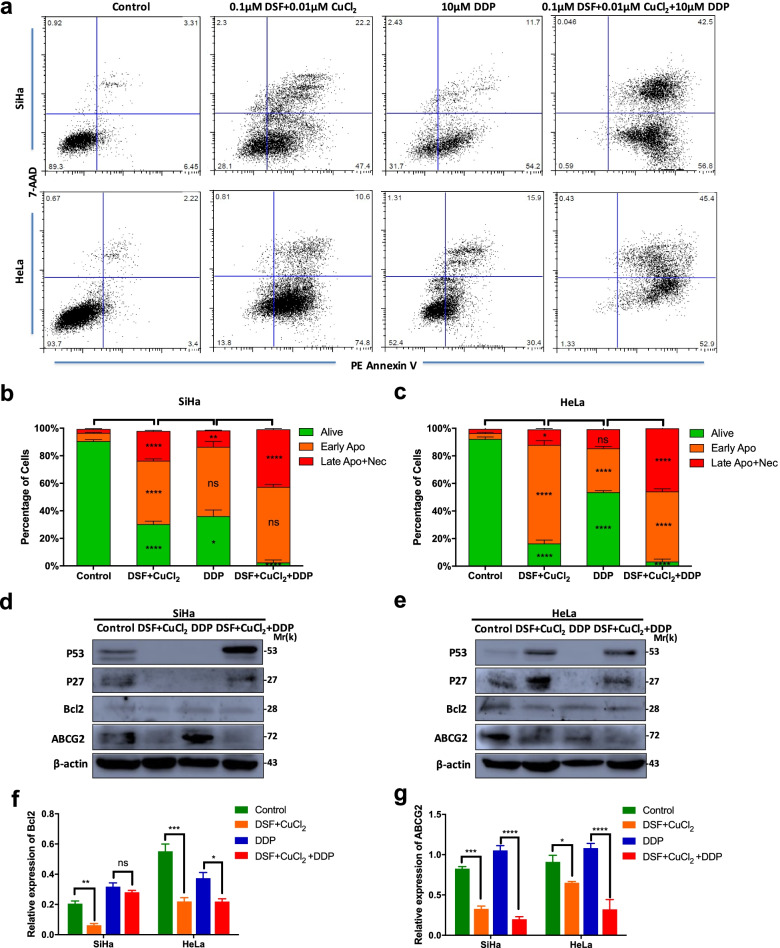
Disulfiram/copper complex induces apoptosis in cervical cancer cell lines. **a** SiHa and HeLa cells were exposed to 10 μM DDP, 0.1 μM disulfiram plus 0.01 μM CuCl_2_ (DSF/Cu) or DSF/Cu plus 10 μM DDP (DSF/Cu/DDP) in combination for 48 h. The drug-treated and control cells were stained with 7-AAD and PE-Annexin V and then analyzed by flow cytometry. **b**-**c** The percentage of different cell populations was identified by 7-AAD/Annexin V assay. **d**-**e** The expression of apoptosis-related proteins and resistance protein was detected by western blot analysis. The full-length blots are presented in Supplementary Fig. 2. **f**-**g** The relative expression levels of bcl2 and ABCG2 were analyzed by Image J programme. Data represent mean ± S.D. of triplicate experiments. *P < 0.05, **P < 0.01, ***P < 0.001, ****P < 0.0001

The apoptotic status of drug-treated cervical cancer cell lines was examined. SiHa and HeLa cells were treated with DSF/Cu complex (0.1 μM DSF + 0.01 μM CuCl_2_), 10 μM DDP or DSF/Cu plus 10 μM DDP (DSF/Cu/DDP) for 48 h. The apoptotic population was detected by flow cytometric with Annexin V/7-AAD staining (Fig. 2a). In comparison with the control or DDP treated groups, a large population of apoptotic cells were detected in DSF/Cu and DSF/Cu/DDP-treated cells, respectively (*P* < 0.01, Fig. 2b-c). The apoptotic results were also confirmed by western blot analysis of some apoptosis-related proteins and resistance protein. DSF/Cu/DDP induced p53 and p27 protein expression in SiHa and HeLa cells. DSF/Cu induced p53 and p27 protein expression in HeLa cells. In addition, DSF/Cu inhibited the expression of bcl2 and ABCG2 protein in two cervical cancer cell lines. The expression of ABCG2 protein in DDP group was significantly higher than DSF/Cu/DDP group in two cell lines (*P* < 0.0001, Fig. 2d-g).

### DSF/cu complex induces the decrease of S-phase in cervical cancer cells

**Fig. 3:**
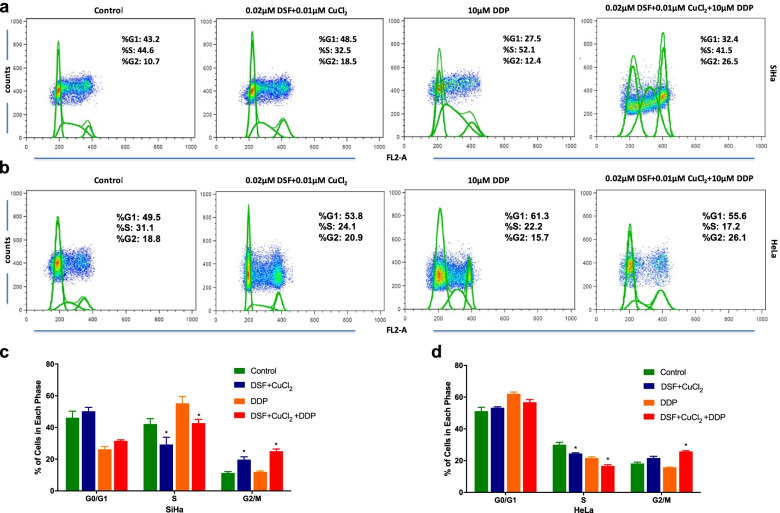
Effect of disulfiram/copper complex on cervical cancer cells through the cell cycle. **a**-**b** SiHa and HeLa cells were treated with 0.02 μM disulfiram plus 0.01 μM CuCl_2_, 10 μM DDP alone or their combination as indicated for 2 days and then followed by cell cycle analysis. **c**-**d** DSF/Cu and DSF/Cu/DDP treatment induced the decrease of cervical cancer cells in S-phase compared with the control and DDP groups, respectively. Data represent mean ± S.D. of triplicate experiments. *P < 0.05

To investigate the mechanism by which DSF/Cu complex inhibits cervical cancer cell proliferation, we analyzed cell cycle status before and after drug treatment. In order to have enough drug action time and avoid premature cell death, the lower concentration of DSF was used. Exposure of SiHa and HeLa cells to 0.02 μM disulfiram plus 0.01 μM CuCl_2_ caused a significant decrease of cells in S-phase relative to control group (both at *P* < 0.05). Consistently, exposure of SiHa and HeLa cells to DSF/Cu/DDP caused a significant decrease of cells in S-phase relative to DDP groups (both at *P* < 0.05). In addition, DSF/Cu treatment led to increased SiHa cells in G2/M phase relative to control group. DSF/Cu/DDP treatment led to increased SiHa and HeLa cells in G2/M phase relative to DDP alone, respectively (*P* < 0.05, Fig. 3a-d). The data suggested that DSF/Cu synergistically inhibited cervical cancer cell proliferation through the decrease of S-phase and G2/M phase arrest.

### DSF/cu complex inhibits the expression of stemness marker in cervical cancer cells

**Fig. 4:**
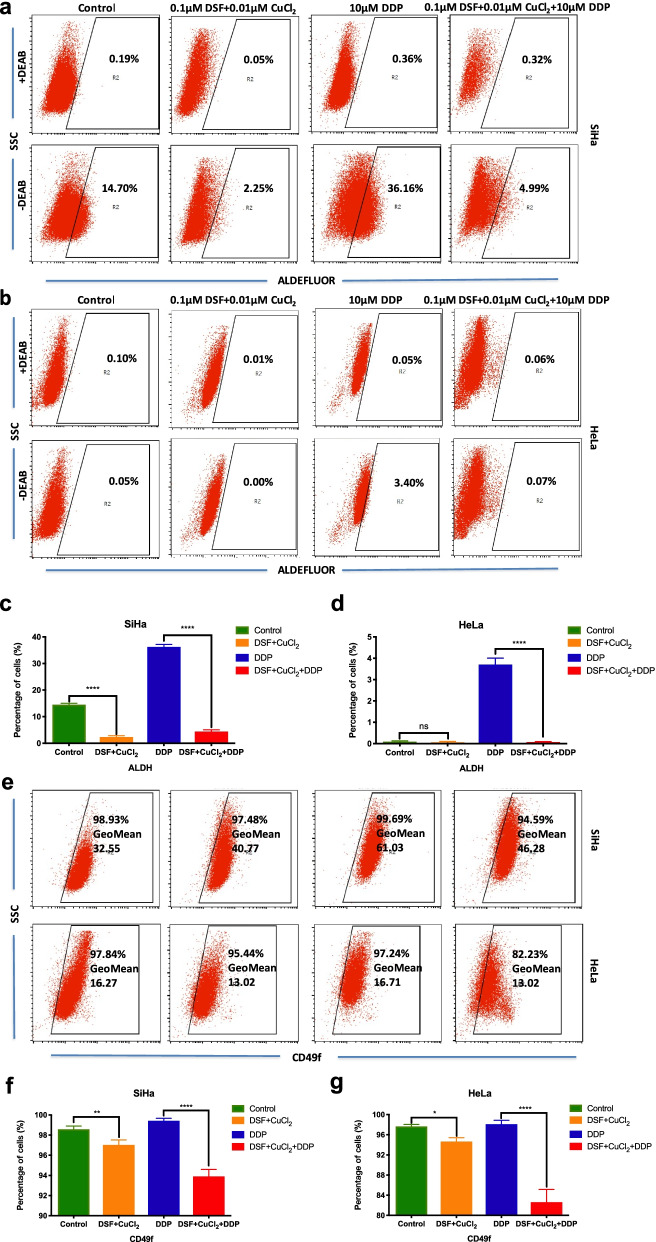
Disulfiram/copper complex inhibits the expression of stemness markers in cervical cancer cell lines. ALDH enzyme activity (**a**-**b**) and CD49f expression (e) in two cervical cancer cell lines were analyzed by flow cytometry after 18 h exposure to different drug and following culture in drug-free culture medium for 3 days. As a negative control for ALDH, the cells were treated with the specific ALDH inhibitor DEAB. The gated cells represent the ALDH-positive cells. Disulfiram/copper complex inhibited the expression of ALDH (**c**-**d**) and CD49f (f-g) in SiHa and HeLa cells. Data represent mean ± S.D. of triplicate experiments. *P < 0.05, **P < 0.01, ***P < 0.001, ****P < 0.0001

It is widely accepted that CSCs are resistant to a wide range of classical cytotoxic anticancer drugs. Our previous study demonstrated that high ALDH activity could represent a functional marker for CCSCs [29]. Disulfiram is a specific inhibitor of ALDH. In this study, we examined the expression of ALDH in DSF/Cu and DDP treated SiHa and HeLa cells. After 18 h exposure to different drugs and following culture in drug-free culture medium for 3 days, a significantly lower percentage of ALDH-positive cells were detected in 0.1 μM disulfiram plus 0.01 μM CuCl_2_ treated SiHa cells than control cells (2.25% vs 14.70%, *P* < 0.0001). In contrast, a significantly higher percentage of ALDH-positive cells were detected in 10 μM DDP treated SiHa cells than control cells (36.16% vs 14.70%, *P* < 0.0001) and DSF/Cu/DDP treated cells (36.16% vs 4.99%, *P* < 0.0001), respectively (Fig. 4a, c). Although there was no ALDH expression in natural HeLa cells, a significantly higher percentage of ALDH-positive cells were detected in DDP treated HeLa cells than DSF/Cu (3.40% vs 0.00%, *P* < 0.0001) and DSF/Cu/DDP treated cells (3.40% vs 0.07%, *P* < 0.0001), respectively (Fig. 4b, d).

We also examined the effect of these compounds on CD49f, another CSC marker. The CD49f expression in DSF/Cu treated SiHa and HeLa cells was significantly reduced compared with their control cells (*P* < 0.05). Consistently, the CD49f expression in DSF/Cu/DDP treated SiHa and HeLa cells was significantly reduced compared with DDP treated SiHa and HeLa cells, respectively (*P* < 0.0001, Fig. 4e-g).

### DSF/cu complex reduces LGR5-positive cervical cancer stem-like cells

**Fig. 5:**
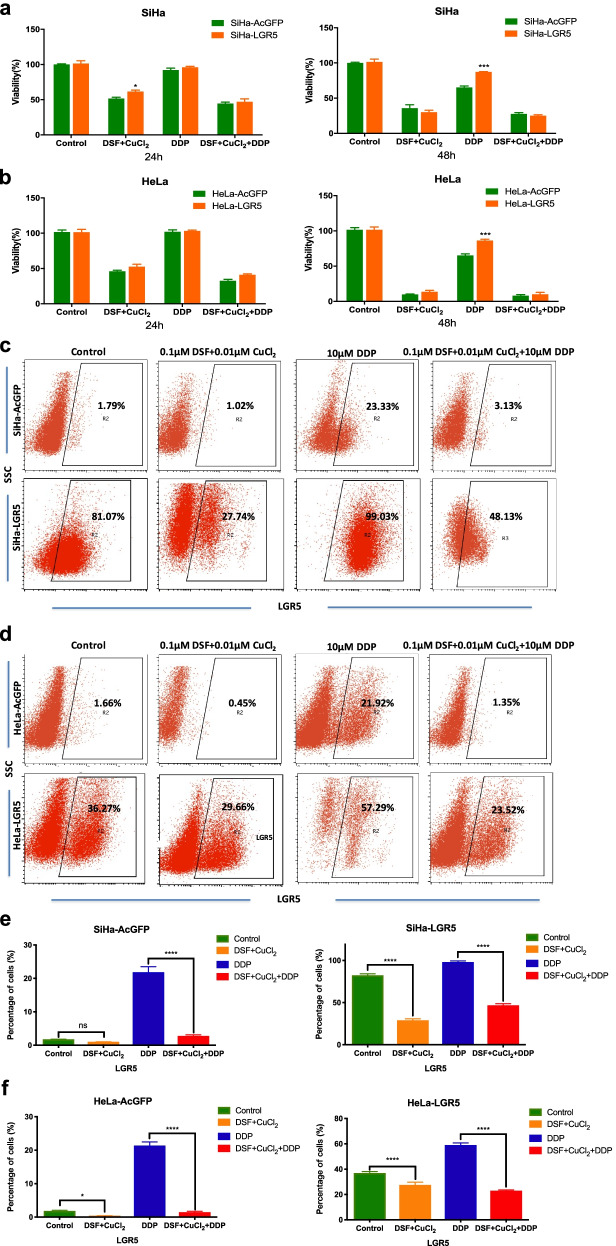
Disulfiram/copper complex reduces LGR5-positive cervical cancer cells. **a**-**b** The LGR5-overexpressing cervical cancer cells and the control cells were treated with 0.1 μM disulfiram plus 0.01 μM CuCl_2_ (DSF/Cu) or 10 μM DDP or DSF/Cu plus 10 μM DDP (DSF/Cu/DDP) for 24 h and 48 h, after which their cell viabilities were determined by the MTT assay. **c**-**d** The percentage of LGR5-positive cells in the cervical cancer cell lines was analyzed by flow cytometry after exposure to different drugs for 3 days and following culture in ordinary culture medium for 2 weeks. **e**-**f** Graphical representation of the statistical analysis of the percentage of LGR5-positive cells in different groups. Data represent mean ± S.D. of triplicate experiments. *P < 0.05, **P < 0.01, ***P < 0.001, ****P < 0.0001

In our study, the DSF/Cu complex strongly inhibited ALDH activity in SiHa cells, which may be a successful therapeutic for cervical cancer by successfully targeting drug-resistant CSC via attenuation of ALDH-mediated protection from damage. Although there was no ALDH expression in natural HeLa cells, the DSF/Cu complex obviously induced apoptosis of HeLa cells. Our previous studies indicated LGR5 promotes cancer stem cell traits and chemoresistance in cervical cancer cells [30]. To further confirm whether the DSF/Cu complex was sensitive to the LGR5-positive cells, stable LGR5-overexpressing cells (SiHa-LGR5 and HeLa-LGR5) were established in cervical cancer cell lines. The expression of LGR5 in these cell lines was examined by immunohistochemistry, western blot and flow cytometry (Supplementary Fig. 1). We tested the effects of DSF/Cu complex and DDP on cervical cancer cells with different expression levels of LGR5. The cell viability was determined using an MTT assay. The cell viability showed time-dependency when these cells treated with different drugs. Both SiHa-LGR5 and HeLa-LGR5 cells were significantly more resistant to DDP than the control cells (SiHa-AcGFP and HeLa-AcGFP, *P* < 0.001), but there was no significantly difference between LGR5-overexpression cells and control cells treated with DSF/Cu or DSF/Cu/DDP (*P* > 0.05, Fig. 5a-b). These results indicated that the DSF/Cu complex produced similar cytotoxicity in LGR5-positive and LGR5-negative cervical cancer cells.

To further confirm whether the DSF/Cu complex were sensitive to the LGR5-positive cells, these cells with different drugs for 3 days and then cultured in regular culture medium for 2 weeks. In the DDP treated group, the percentage of LGR5^+^ cells expanded from 1.79 to 23.33% in the SiHa-AcGFP cell population, 81.07 to 99.03% in the SiHa-LGR5 cell population, 1.66 to 21.92% in the HeLa-AcGFP cell population and 36.27 to 57.29% in the HeLa-LGR5 cell population. However, in the DSF/Cu treated group, the percentage of LGR5^+^ cells reduced from 1.79 to 1.02% in the SiHa-AcGFP cell population, 81.07 to 27.74% in the SiHa-LGR5 cell population, 1.66 to 0.45% in the HeLa-AcGFP cell population and 36.27 to 29.66% in the HeLa-LGR5 cell population. Additionally, in the combination group of DSF/Cu/DDP, the percentage of LGR5^+^ cells was significantly lower than DDP group (*P* < 0.0001, Fig. 5c-f). These data suggested that the DSF/Cu complex could reduce LGR5-positive cervical cancer cells which had the ability of resistance to cisplatin treatment.

### DSF/cu complex inhibits LGR5-positive cervical cancer cells in vivo

**Fig. 6:**
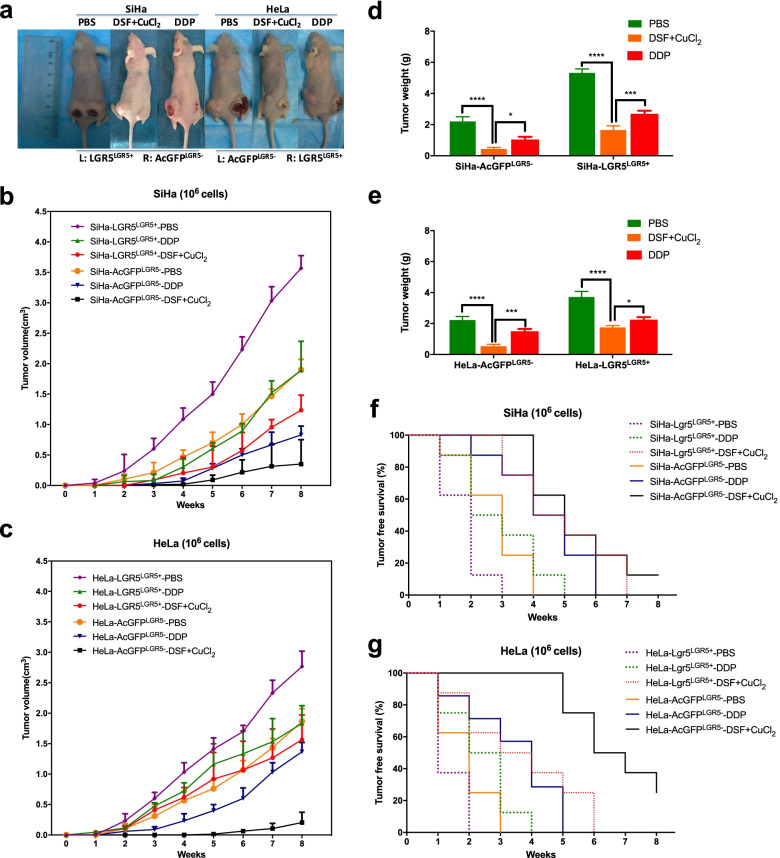
Disulfiram/copper complex inhibits LGR5-positive cervical cancer cells in vivo*.* **a** Suppression of tumor growth after DSF/Cu and DDP treatment in female BALB/c-nude mice. **b**-**c** The volume of xenograft tumors formed by LGR5^LGR5+^ and AcGFP^LGR5-^ cervical cancer cells with different drug delivery was monitored over time. **d**-**e** At the end of the experiment, the tumor weights were measured. **f**-**g** Kaplan-Meier plots showing the tumor-free survival after injection. Data represent mean ± S.D. of tumor volumes or weights of eight mice in each group. *P < 0.05, **P < 0.01, ***P < 0.001, ****P < 0.0001

To determine whether DSF/Cu can inhibit LGR5-positive cervical cancer stem-like cells in vivo, we used a xenograft model of modified LGR5-positive/negative SiHa and HeLa cells in BALB/c mice (Fig. 6a). Tumor growth curves are shown in Fig. 6b and c. The tumors formed by the LGR5^+^ cells were larger and grew faster than those formed by the LGR5^−^ cells in each group (*P* < 0.05), but the tumors in the DSF/Cu treated group were significantly smaller and slower than those treated with DDP and PBS in both modified SiHa and HeLa cell groups (*P* < 0.05). Upon completion, tumor weight for treatment groups of DSF/Cu was smaller than those treated with DDP and PBS groups (Fig. 6d-e). Tumor latency was monitored after the injection of sorted cells into BALB/c mice and was defined the tumor-free duration in the mice. During 8 weeks, the AcGFP^LGR5-^ cell groups treated with DSF/Cu caused the higher tumor-free survival rates (12.5% for SiHa-AcGFP^LGR5-^ cell group and 25% for HeLa-AcGFP^LGR5-^ cell group versus 0% for other groups) than DDP groups and control groups. (*P* < 0.05, Fig. 6f-g). These data indicated that the treatment with DSF/Cu complex had the greater efficacy in tumor growth inhibition compared with DDP groups whether in LGR5-positive or LGR5-negative cervical cancer cells in vivo.

## Discussion

The tumor originates from monoclonal cell and appears to contain a heterogeneous population of tumor cells. According to the cancer stem cell hypothesis, among these heterogeneous cell populations, only a few subpopulation of cancer cells, possess enhanced self-renewal capacity, differentiation potential, tumorigenicity and chemoresistance [32]. Our previous studies have demonstrated that high aldehyde dehydrogenase (ALDH) activity may represent a functional marker for cervical CSCs and LGR5 can promote cervical cancer stem cell traits and chemoresistance [29, 30]. Here, we explored to find a drug targeting CSCs for cervical cancer therapies. It has been observed that the patients who continuously used DSF (an ALDH inhibitor) have a lower risk of death from cancer compared with those who stopped using DSF at their diagnosis in an epidemiological study [33]. DSF has also been shown to be effective against some cancer types in preclinical studies. However, little is known about the mechanisms and therapy of DSF in cervical cancer. In the present study, we show, for the first time, the cytotoxicity of disulfiram may be superior to cisplatin caused by targeting LGR5-positive cervical cancer stem-like cells in cervical cancer.

In this study, DSF with exogenous Cu^2+^ supplementation exhibited dose-dependent and time-dependent cytotoxicity in cervical cancer cells, but disulfiram alone had no therapeutic effect in vitro. Researchers reported recently demonstrated that DSF/Cu treatment eliminated the stem cell-like ALDH ^+^ cells pool in non-small cell lung cancer [34, 35]. Our previous study showed that the ALDH^high^ cells were more resistant to cisplatin treatment than the ALDH^low^ cells in cervical cancer [29]. In the study, the proportion of ALDH positive cells increased in SiHa and HeLa cells after cisplatin treatment (Fig. 4a-d), which may be because ALDH-positive cells can survive and reconstitute the cellular hierarchy. Excitedly, we found that the DSF/Cu complex alone or in combination with DDP could also eliminate ALDH^+^ cell population in SiHa cells and DDP treated HeLa cells. Interestingly, although there is no ALDH expression in untreated HeLa cells, the DSF/Cu complex induced more remarkable cell apoptosis and exhibited greater efficacy of tumor growth inhibition on HeLa cells compared with DDP in vitro and in vivo. Our previous studies manifested that overexpression of LGR5 promotes cervical cancer cell stemness and chemoresistance [30]. According to this study, the DSF/Cu complex exhibited equivalent effect whether LGR5-positive cervical cancer stem-like cells or LGR5-negative cells both in vitro and in vivo systems. In other words, DSF/Cu treatment was superior to cisplatin by its ability of eliminating the stem cell-like LGR5 ^+^ cells pool in cervical cancer. As shown in the Fig. 5, after the cells were killed for 3 days and then cultured in regular culture medium for 2 weeks, the expression of LGR5 in the surviving LGR5-overexpressed cells was significantly lower than that in the control group. These data indicated that DSF/Cu might cause the persistent down-regulation of LGR5 in cervical cancer for eliminating LGR5 ^+^ cells pool.

Cisplatin is one of the most potent antitumor agents known, and its cytotoxic mode of action is mediated by activating several signal transduction pathways, including ATR, p53, p73 and MAPK. However, its resistance mechanisms include loss of damage recognition, loss of p53 function and overexpression of antiapoptotic bcl-2 [36]. It has been confirmed that DSF increases the level of p53 and induced p53-dependent apoptosis [37]. In the study, the expression of p53 and p27 was significantly decreased in DDP treated SiHa and HeLa cells, but increased in DSF/Cu treated HeLa cells and DSF/Cu/DDP treated SiHa and HeLa cells compared to control groups (*P* < 0.05, Fig. 2). In addition, bcl-2, an anti-apoptotic protein, has been reported to regulate the apoptotic pathways and protect against cell death [38, 39]. The multidrug resistance gene ABCG2 is also an important proliferation-promoting oncogene in cervical cancer [40]. We also observed that DSF/Cu treatment resulted in a marked decrease of bcl-2 and ABCG2 protein expression in SiHa and HeLa cells, which was superior to that of cisplatin (Fig. 2f-g). However, in detail, the changes of these genes caused by DSF/Cu and combination group are asynchronous in HeLa and SiHa cells, which need further study.

It has been reported that DSF/Cu treatment causes accumulation of cells in G2 and inhibits DNA synthesis [18]. In our study, the decrease in the proportion of S phase following DSF/Cu treatment was accompanied by an increase in the proportion of G2 phase in SiHa cells. Although the increase in the proportion of G2 phase following DSF/Cu treatment was not observed in HeLa cells, both SiHa and HeLa cells were corroborated the observation that DSF/Cu/DDP caused arrest in G2. When DNA damage occurs in the G2 phase, the expression levels of p53 and p27 are increased, cells undergo G2/M phase cell cycle arrest, p53 and its downstream gene p27 promotes apoptosis [41–43]. In our study, these changes were consistent only in the combination group in SiHa and HeLa cells, which indirectly reflected that DSF/Cu induces the changes of cell cycle and apoptosis through different molecular models in SiHa and HeLa cells.

Previous studies developed a vaginal tablet of DSF to be used in HeLa and Ca-Ski cells for the localized treatment of cervical cancer [28, 44]. This study showed animal experiments provided a basis for systemic application of DSF. In addition, the DSF/Cu complex could inhibit the expression of stemness markers and suppress the cisplatin-resistant LGR5^+^ stem-like population in cervical cancer cells. It is attractive to consider DSF as an independent treatment or adjuvant treatment combined with cisplatin to establish a new chemotherapy protocol for targeting cervical CSCs. However, more research is still needed to further explore the effect of DSF on primary tumors from patients.

## Conclusion

Taken together, these results indicate that the disulfiram/copper complex is toxic in cervical cancer cell lines in vitro and in vivo by mechanisms including targeting LGR5^+^ cancer stem-like cells. We encourage for further clinical trials in cervical cancer with DSF, an old, safe and public domain drug [4].

## Supplementary Information

**Additional file 1: Supplement Figure 1.** Overexpression of LGR5 in human cervical cancer cell lines. (a-b) Immunocytochemistry staining showing LGR5 expression in LGR5-overexpressing SiHa and HeLa cells, scale bar, 10 μm. (c-d) LGR5 expresssion was analyzed by flow cytometry. (e) A western blot assay was used to characterize the expression of LGR5 in LGR5-overexpressing SiHa and HeLa cells. The full-length blots are presented in Supplementary Fig. 3. (f) The expression levels of LGR5 in HeLa and SiHa cells were measured by western blot. AcGFP: green fluorescent protein for control; LGR5: overexpression for LGR5. Values are shown as the mean ± S.D. **P < 0.01.

**Additional file 2: Supplementary Figure 3.** The full-length blots of LGR5 in LGR5-overexpressing SiHa and HeLa cells. The blots were cutted according to molecular size markings prior to hybridisation with antibodies.

**Additional file 3: Supplementary Figure 2.** The full-length blots of apoptosis-related proteins and resistance protein was detected by western blot analysis. The blots were cutted according to molecular size markings prior to hybridisation with antibodies.

## Data Availability

The datasets used and/or analyzed during the current study are available from the corresponding author on reasonable request.
