# Supplementary figures and images for: Cytotoxic effect of disulfiram/copper on human cervical cancer cell lines and LGR5-positive cancer stem-like cells
Source: BMC Cancer. 2022 May 9;22:521. doi: 10.1186/s12885-022-09574-5 (PMC9082913; doi:10.1186/s12885-022-09574-5)

**a**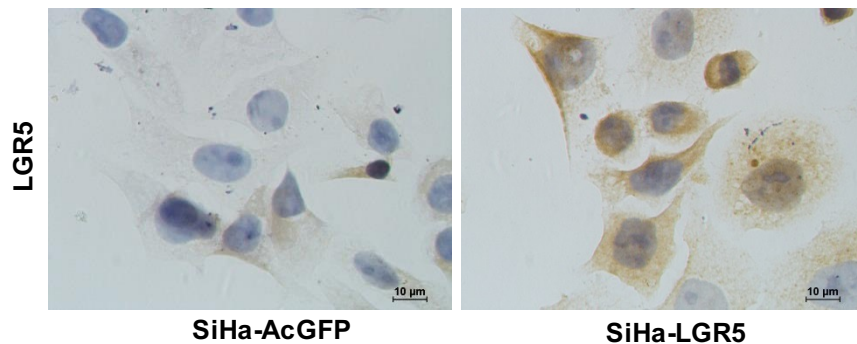**b**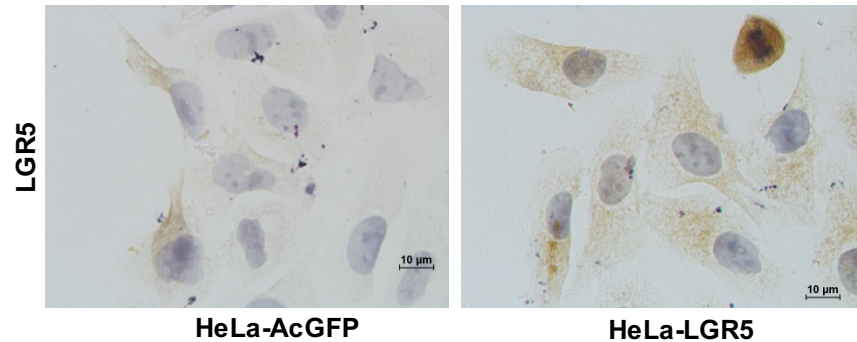**e**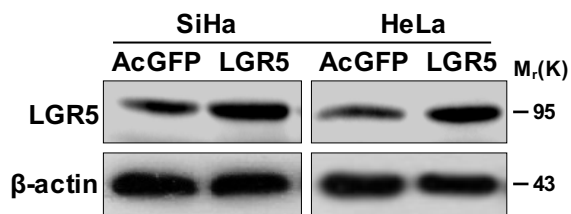**f**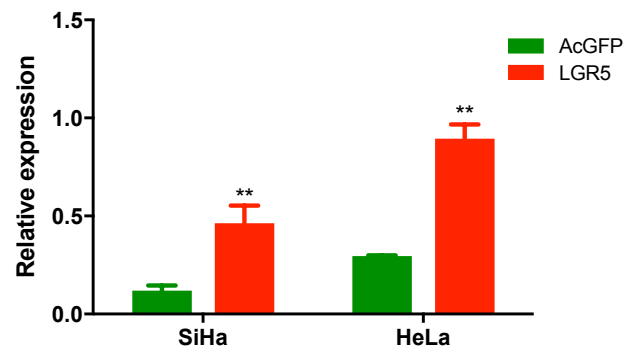**c**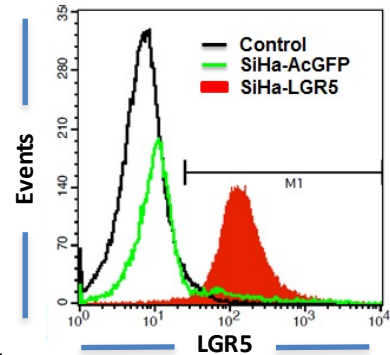**d**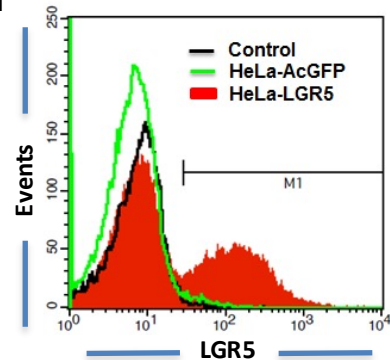

Supplement: Supplementary file 1 — Additional file 1: Supplement Figure 1. Overexpression of LGR5 in human cervical cancer cell lines. (a-b) Immunocytochemistry staining showing LGR5 expression in LGR5-overexpressing SiHa and HeLa cells, scale bar, 10 μm. (c-d) LGR5 expresssion was analyzed by flow cytometry. (e) A western blot assay was used to characterize the expression of LGR5 in LGR5-overexpressing SiHa and HeLa cells. The full-length blots are presented in Supplementary Fig. 3. (f) The expression levels of LGR5 in HeLa and SiHa cells were measured by western blot. AcGFP: green fluorescent protein for control; LGR5: overexpression for LGR5. Values are shown as the mean ± S.D. **P < 0.01. [file 12885_2022_9574_MOESM1_ESM.pdf]

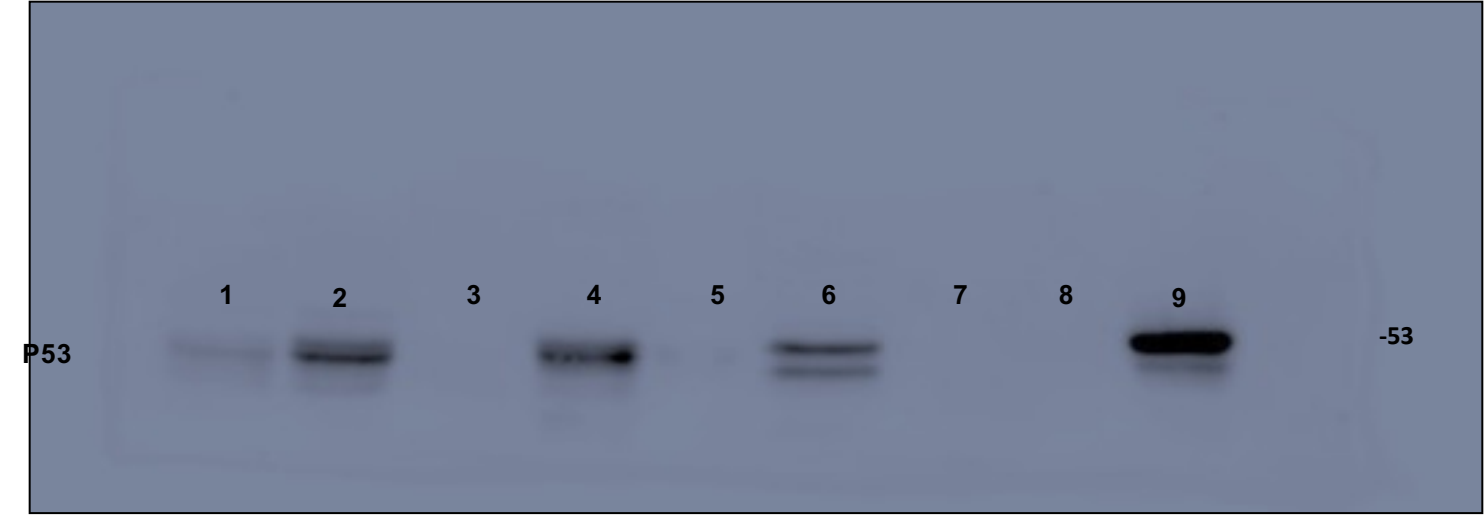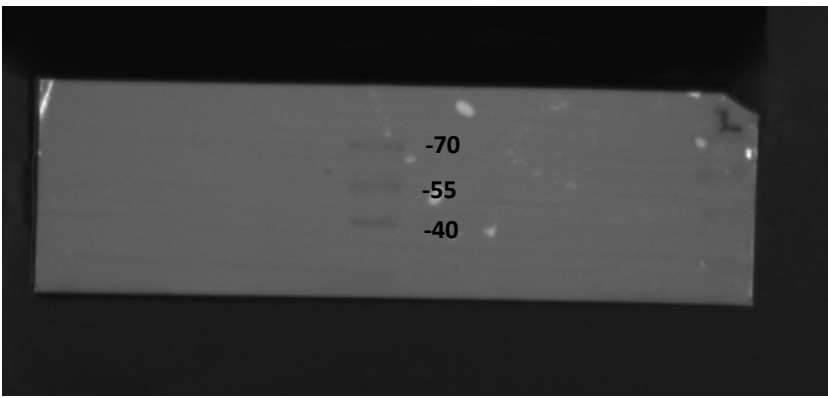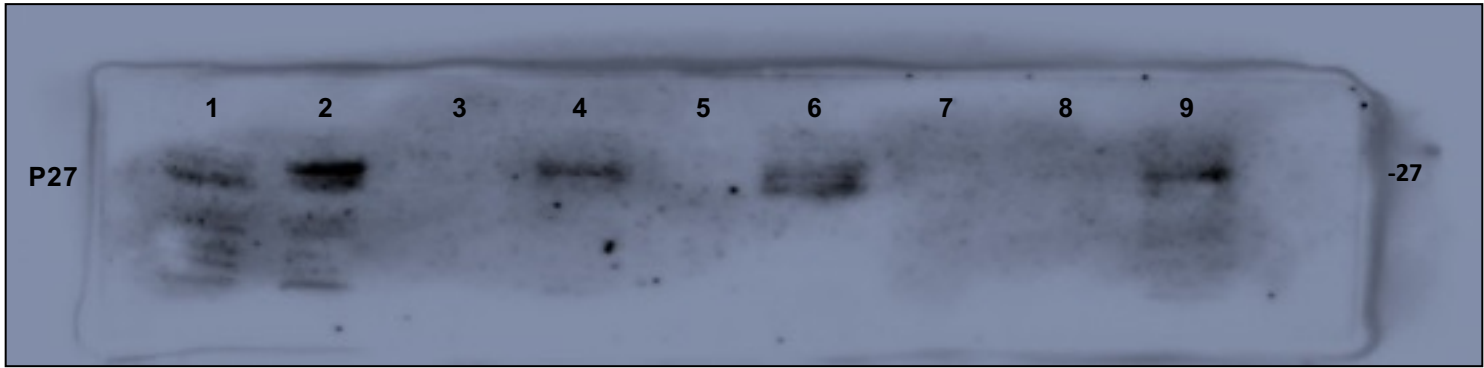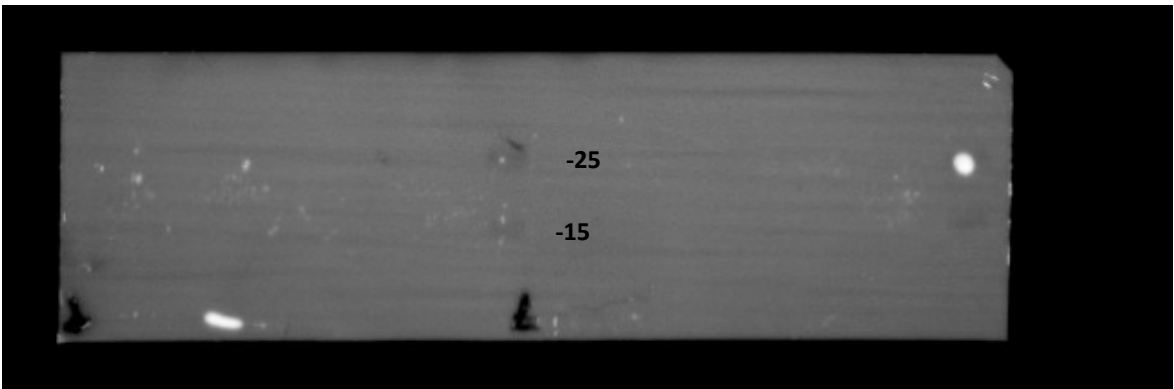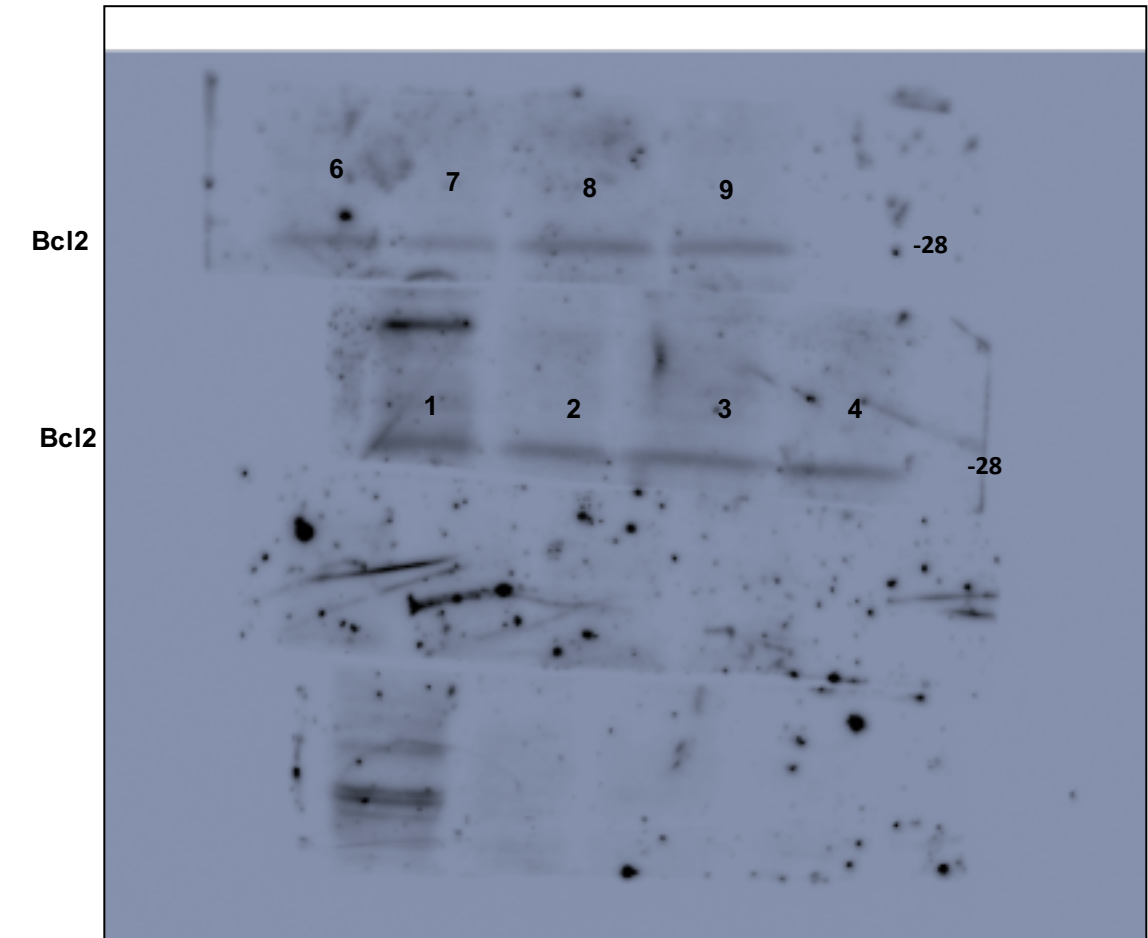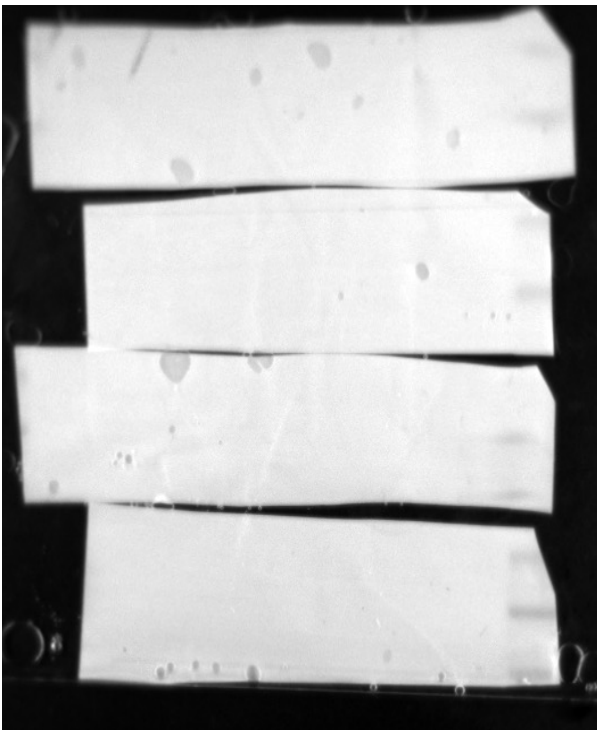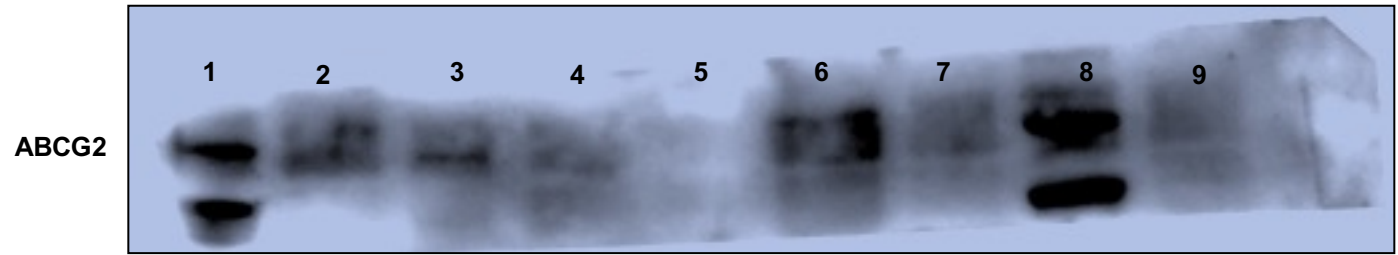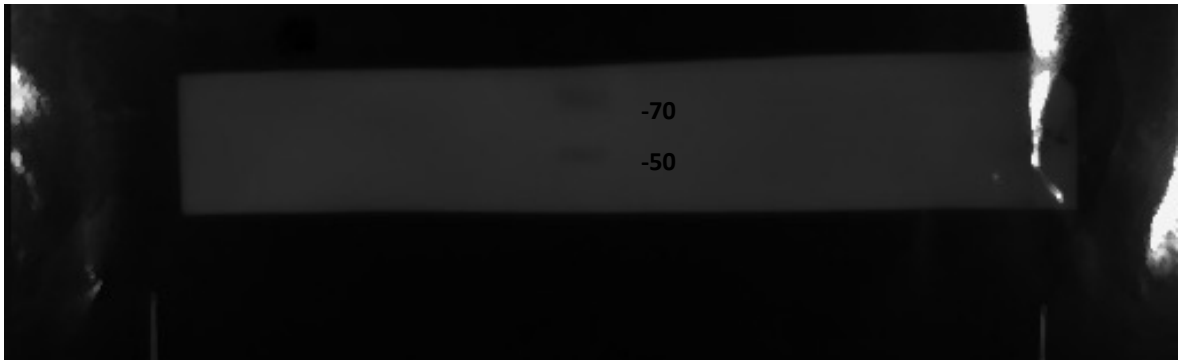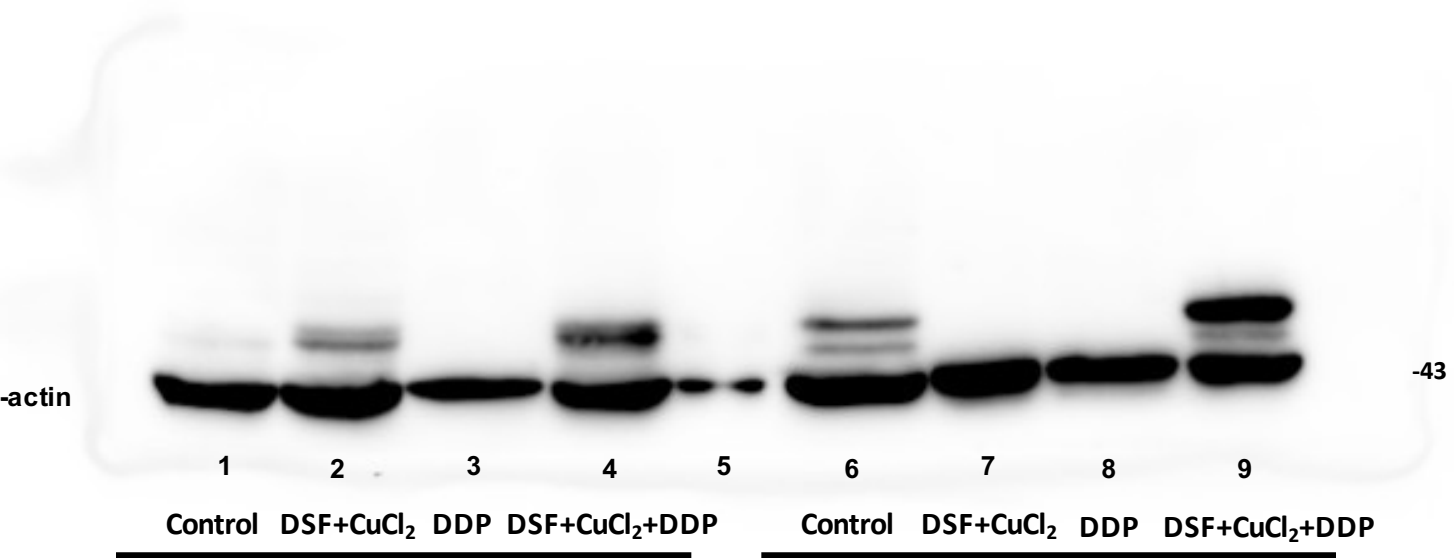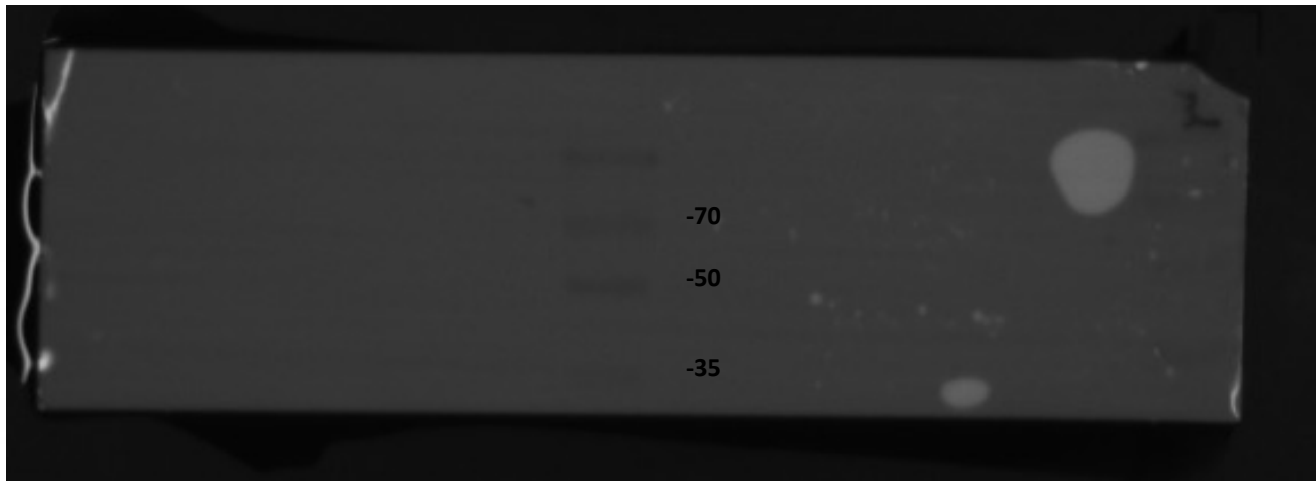

HeLa  
(Fig. 2 e)

Marker

SiHa  
(Fig. 2 d)

Marker

Supplement: Supplementary file 2 — Additional file 2: Supplementary Figure 3. The full-length blots of LGR5 in LGR5-overexpressing SiHa and HeLa cells. The blots were cutted according to molecular size markings prior to hybridisation with antibodies. [file 12885_2022_9574_MOESM2_ESM.pdf]

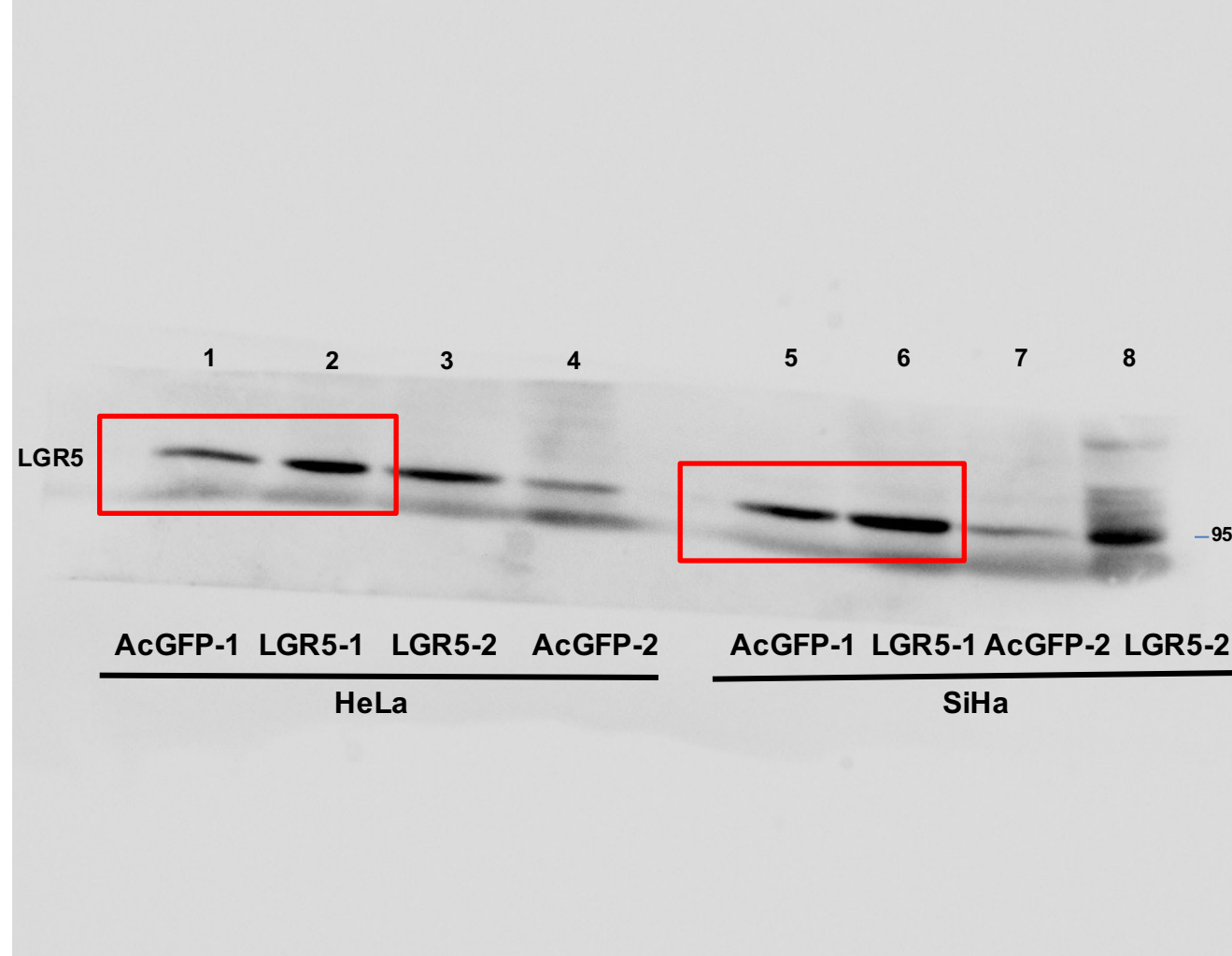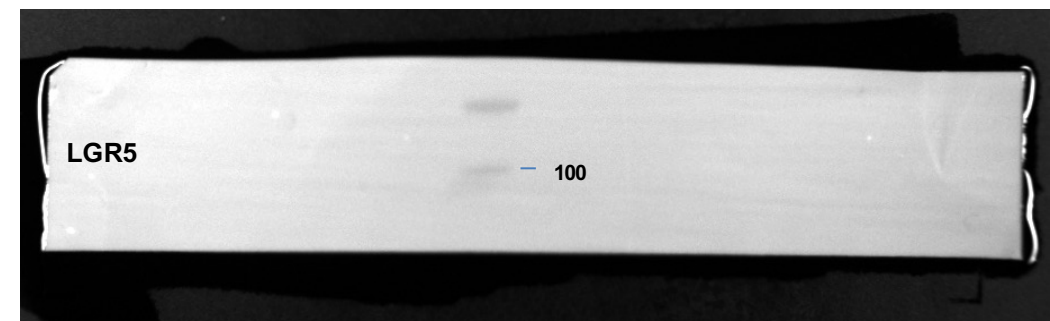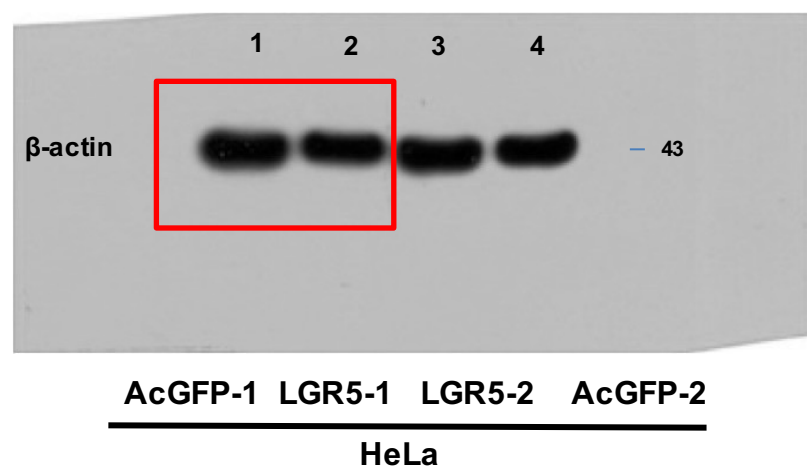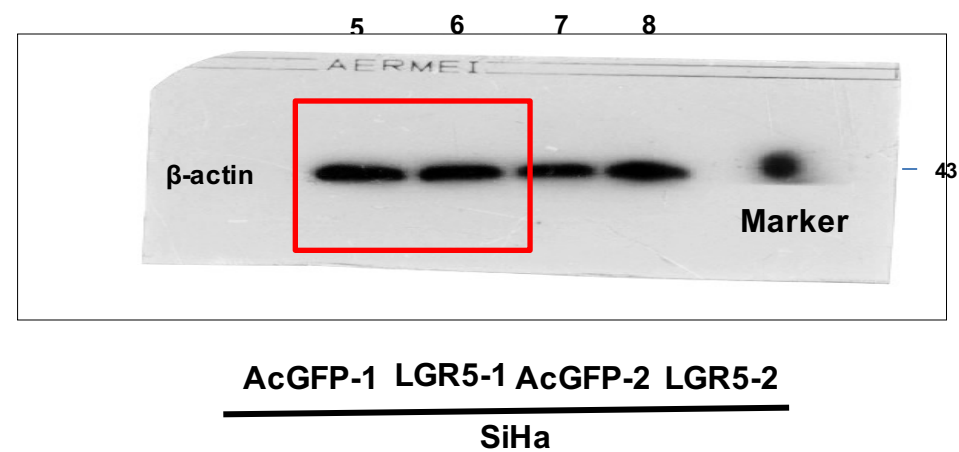

(Supplementary Fig. 1e)

Supplement: Supplementary file 3 — Additional file 3: Supplementary Figure 2. The full-length blots of apoptosis-related proteins and resistance protein was detected by western blot analysis. The blots were cutted according to molecular size markings prior to hybridisation with antibodies. [file 12885_2022_9574_MOESM3_ESM.pdf]
